# Trinucleotide Repeats and Haplotypes at the Huntingtin Locus in an Indian Sample Overlaps with European Haplogroup A

**DOI:** 10.1371/currents.hd.a3ad1a381ab1eed117675145318c9a80

**Published:** 2014-09-24

**Authors:** Nagaraj S Moily, Lakshmi Narayanan Kota, Ram Murthy Anjanappa, Sowmya Venugopal, Radhika Vaidyanathan, Pramod Pal, Meera Purushottam, Sanjeev Jain, Mahesh Kandasamy

**Affiliations:** Molecular Genetics Laboratory, Department of Psychiatry, National Institute of Mental Health and Neurosciences (NIMHANS), Bangalore, India; The University of Melbourne, Melbourne,Australia; Molecular Genetics Laboratory, Department of Psychiatry, National Institute of Mental Health and Neurosciences (NIMHANS), Bangalore, India; Human Molecular Genetics, Molecular Biology and Genetics Unit, Jawaharlal Nehru Centre for Advanced Scientific Research, Bangalore, India; Molecular Genetics Laboratory, Department of Psychiatry, National Institute of Mental Health and Neurosciences (NIMHANS), Bangalore, India; Molecular Genetics Laboratory, Department of Psychiatry, National Institute of Mental Health and Neurosciences (NIMHANS), Bangalore, India; Department of Neurology, National Institute of Mental Health and Neurosciences (NIMHANS), Bangalore, India; Molecular Genetics Laboratory, Department of Psychiatry, National Institute of Mental Health and Neurosciences (NIMHANS), Bangalore, India; Molecular Genetics Laboratory, Department of Psychiatry, National Institute of Mental Health and Neurosciences (NIMHANS), Bangalore, India; Molecular Genetics Laboratory, Department of Psychiatry, National Institute of Mental Health and Neurosciences (NIMHANS), Bangalore, India

## Abstract

Huntington’s disease (HD), an autosomal dominant neurodegenerative syndrome, has a world-wide distribution. An estimated 2.5-10/100,000 people of European ancestry are affected with HD, while the Asian populations have lower prevalence (0.6-3.8/100,000). The epidemiology of HD is not well described in India, and the distribution of the pathogenic CAG expansion, and the associated haplotype, in this population needs to be better understood. This study demonstrates a distribution of CAG repeats, at the HTT locus, comparable to the European population in both normal and HD affected chromosomes. Further, we provide an evidence for similarity of the HD halpotype in Indian sample to the European HD haplogroup.

## Introduction

The diagnosis of Huntington’s disease (HD) is based on estimation of the CAG repeat length at the HTT locus 1. The normal HTT gene contains less than 27 CAG repeats 2 ^,^ 3 , and a few normal individuals have intermediate CAG_(27-35) _repeat expansion 2 and display no symptoms suggestive of HD. Subjects with borderline CAG_(36–39) _repeats may or may not develop symptoms. Individuals affected with HD typically have at least one HTT allele containing CAG repeat size of 40 or greater 2 ^,^ 4.

The age at onset (AAO) is inversely correlated with length of the pathogenic CAG stretch in the HTT gene 5 . Almost 50-70% of the variation observed is determined by the CAG repeat length, the remaining maybe explained by the additional influence of other cis and trans elements, as well as environmental factors^5^ . Highly expanded CAG sequences cause disease onset at a younger age 6 . The fundamental mechanisms of CAG repeat instability are poorly understood.

The prevalence of HD varies among different populations, with prevalence rates of 2.5 -10 per 100,000 in people of European ancestry, while the Japanese (0.11–0.45 per 100,000), Chinese ( 0.5-1 per 100,000) and African populations (<0.01 per 100, 000) show significantly lower prevalence^7^ . Indian and other South Asian populations are expected to have intermediate prevalence of HD. Prevalence studies of Indian immigrants in UK, predominantly from the northern regions of the Indian subcontinent 8 suggest that HD occurs in 1.75 per 100,000 individuals. It is generally accepted from clinical experience, and family studies of different geographical regions, that HD is distributed widely in India 9 ^,^ 10 ^,^ 11 ^,^ 12 . The origin of the pathogenic CAG expansion in India is not well understood. Multiple founder effects, and admixture with European populations probably has caused the spread of HD into diverse populations across the world^9^^,^ 10 ^,^ 13 .

Pramanik *et al* 10 reported CAG repeat expansions in 28 HD samples from eastern part of India ranging from 41-56 while the range of the normal allele on the homologous chromosome was 13–29 repeats. In a previous study 11 of 117 individuals we reported that the mean CAG repeats on normal alleles was 18±2.4 with a range of 14–30, and in the 26 subjects with HD, the mean CAG repeat length in expanded alleles was 48.4 ± 8.7 with the range of 39 to 82. Using another marker (CCG repeats) adjacent to the CAG repeats in the HTT gene, it has been observed that (CCG)_10 _repeat size was linked to the majority (84.5%) of expanded alleles in the Japanese populations, while in the Caucasian populations (CCG)_7 _repeat was associated with the expanded alleles^10^^,^ 11 ^,^ 12 . There was no significant correlation found between CCG repeats and expanded CAG in Indian samples 10 .

A polymorphic ∆2642 (GAG insertion/deletion) of the HTT locus indicated that majority of the insertion (i.e. the presence of GAG) is associated with the normal alleles while deletion is associated with the pathological expansions 14 . In addition a marker D4S127 (CA repeat allele 2 -151bp) has significantly been associated with HD patients originated from the southern part of India, while this was not seen in HD patients originated from northern India.

Recent reports suggests that differences in the prevalence of HD may be linked to differences in haplotypes 3 ^,^ 7 . Warby et al 3 hypothesized that HTT haplogroups (A, B and C) are associated with differences in mutational rates, thereby influencing the prevalence across populations. Haplogroup A is largely seen in HD patients from Europe, while in East Asian populations (China and Japan), HD alleles are associated with haplogroup C. The HTT haplotypes in Indian population are not determined yet. In this study, we describe the genetic characteristics of the HD mutation and the prevalence of the common HTT haplotypes in the Indian population.

## Methodology

**Enrolment of Clinical Samples:**

The clinical samples (N=164) were derived from both outpatients and inpatient referrals to Genetic Counseling and Testing Center (GCAT) at the National Institute of Mental Health and Neurosciences (NIMHANS), Bangalore, India. The healthy control samples (N=103) were recruited by purposive sampling of volunteers from different parts of India (Total, N=267). Written informed consent was obtained after detailed explanation and genetic counseling of all the subjects and family members prior to enrollment into the study. This study was approved by the Institutional Ethics Committee, National Institute of Mental Health and Neurosciences, (NIMHANS) Bangalore, India.

**Genotyping of CAG repeats CCG repeats,** **∆2642 and D4S127 polymorphisms at HTT locus:**

The genomic DNA was extracted from whole blood using standard protocols 15 . The extracted DNA was subjected to polymerase chain reaction (PCR) using appropriate primers. In patients and normal chromosomes, the CAG repeats, CCG,**∆**2642 and D4S127 polymorphisms were analyzed as described previously 14 . The PCR products were characterized by high resolution agarose gel electrophoresis and fragment analysis was performed using ABI 3500xL genetic analyzer. Repeat sizing was done using Gene Mapper v3.5. PHASE 17 was used to determine the most likely haplotype for the samples.

**Analysis of Haplogroup A, B C in HD cases**

Of the reported twenty-two predisposing tag SNPs 3 , three SNPs (rs762855 (SNP1), rs3856973 (SNP2) and rs4690073 (SNP3) were selected to distinguish haplogroups A, B and C. The PCR was carried out by designing appropriate sets of primers. The SNP1 (rs762855) was determined by Sanger’s sequencing in ABI 3500xL genetic analyzer. The SNP2 and SNP3 were detected by PCR RFLP using restriction enzymes TaqI and AseI respectively.

**Statistical analysis**

The mean CAG repeat numbers was compared by Mann-Whitney U test using R software and QTI- plot 16 , and statistical significance was defined as P<0_._05. Spearman’s rank correlation and partial correlation coefficients were used for correlation analysis.

## Results

**Description of the clinical sample:**

Of 164 clinical samples, pathological expansion of more than 39 CAG repeats was confirmed in 116 (71%) samples (symptomatic, N=102, and pre-symptomatic, N=14). The remaining samples (N=48, 29%) had choreiform movements, but did not show any CAG expansion at the HTT locus.

Almost half of the confirmed sample showed paternal inheritance (N= 46; 45.1%), while maternal inheritance (N=25; 24**^.^**5%) was seen in a quarter. Both parents were affected in two (1.9%) cases. ‘Sporadic’ mutations without any reported family history were seen in 11 (11%) cases. Inheritance information was not available for 18 cases (17.6%) (Fig 1A).

**Distribution of CAG repeats at HTT locus**

The control group showed a mean CAG distribution of 17.6± 2 (range: 11- 34) in the lower allele (LA) and 20.1±3.6 (range: 15- 34) in the upper allele (UA) (Fig. 1B,C D). The symptomatic HD cases (N=102) showed a mean distribution of 17.9±2.8 in LA and 45.9±7.3 in UA (Fig. 1B,C,D). There were 14 pre-symptomatic individuals with expanded alleles. (LA= 17.6±1.7; UA=42.9±3.1; range 36-48).

The distribution of LA among the HD cases and healthy controls (Fig 1B) did not differ significantly (Control:17.6; HD:17.9). The number of CAG repeats in HD sample ranged from 40 to 85 repeats in the UA. Expansions between 40-50 repeats were most common (N=86, 84.3%), with expansions greater than 50 CAG repeats being less frequent (N=16, 15.7%) (Fig1 D).

**Mode of inheritance and distribution of CAG repeats in the study group and Distribution of (CAG)n size of the Huntington disease (HD) gene in 438 chromosomes of the Indian population.**
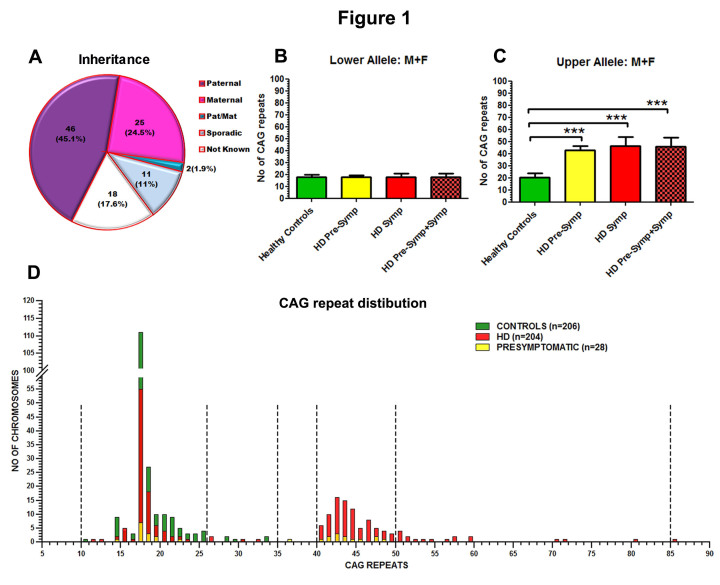
Fig.1A: Mode of inheritance based on detailed clinical interviews of symptomatic HD individuals and family members. Number of individuals (%) Fig.1B: Distribution of CAG repeats at the lower allele (LA) in healthy controls, HD symptomatic and pre symptomatic individuals. Fig.1C: Distribution of CAG repeats at the upper allele (UA) in healthy controls, HD symptomatic and pre symptomatic individuals. *** p≤0.001 Fig.1D: The CAG distribution in the Indian population shows a pattern of 5 distinct spreads.

The average AAO was 37.6±12 years, with the CAG repeat size correlating inversely with the AAO (r = -0.7 P<0.0001) (Fig.2), and accounted for about 51.4% of the variance at onset. HD individuals with CAG 40-50 repeats had an AAO of 40.2±10.4 years, while those with more than 50 CAG repeats had a much lower AAO (19.5± 5.0) (r = -0.96 P<0.0001). In these subjects, CAG repeats accounted for about 93.3% of the variance at onset (Fig. 2). Notably, the highest CAG repeat size (85 repeats) was seen in a child aged 4 years.

**Inverse correlation of AAO and HD CAG repeat length.**
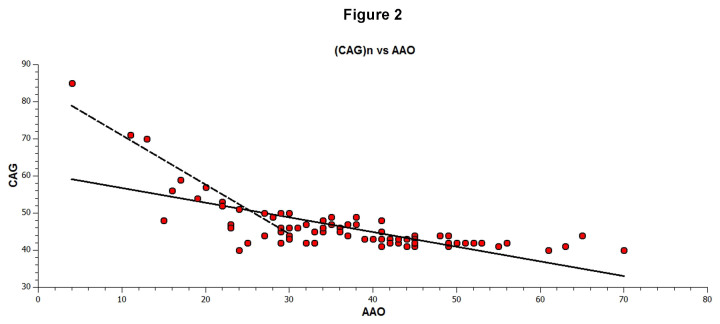
For each individual, the measured CAG repeat length in HTT allele (Y-axis) is plotted against AAO (years) (X-axis). The solid line represents the linear fit to the data. The CAG repeat length accounts for approximately 51.4% of the overall variation in AAO. The dotted line shows the linear fit for repeats >50. The CAG repeat length accounts for approximately 93.3% of the overall variation in AAO.

**Comparison of CAG repeats in control and HD cases from different geographical regions of India:**

The healthy control group had 88 individuals of southern Indian descent (LA= 17.6±2.1; UA= 19.8±3.5) and 15 individuals from northern and eastern parts of India (LA=18.0±1.3; UA=21.6±3.9). In the symptomatic HD sample (N=102), 75 were of southern Indian origin (LA= 17.8±3.1; UA= 46.5±7.6; AAO 37.8±11.7) and 27 were from the northern and eastern parts of India (LA= 18.3±2.3; UA=45.8±7.8; AAO 37.2±13.2). The distribution of CAG repeats did not differ in number of CAG repeats, or in the AAO, between these regions.

**Distribution of CCG repeats, ∆2642 and D4S127 polymorphisms at the HTT locus in controls and HD patients:**

The (CCG)_7 _repeat length was the most frequent followed by the (CCG)_10 _repeat length regardless of genotype. The (CCG)_10 _repeat size had a much lower frequency in HD cases compared to controls. The Δ2642 marker was found in 90% of the HTT alleles corresponding to 'In' (insertion) polymorphism in both cases and controls. In the remaining 10% of subjects, this Δ2642 marker was absent (deletion) in 8% of cases compared to 1.7% of controls. Moreover, more than one third of high end normal alleles had this deletion. The D4S127 microsatellite repeat distribution was similar with 5(153bp) being the most common allele in both cases and controls. As parental genotypes could not be ascertained, PHASE 17 was used to ascertain the most probable haplotype for these 3 markers. Both cases and controls showed the 7-In-5(153bp) to be the most likely combination.

**Distribution of haplogroup A, B and C in HD patients:**

Due to the non availability of all parental DNA samples and genotype information, it was difficult to establish haplotype A, B, C for all samples. However, we found that out of 90 samples with expanded CAG repeats which had genotype data available, those that were homozygous (60%) for these three SNPs, had the AGG haplotype. Thus region wide analysis showed 62.8% of the southern Indian, 60% of the northern Indian and 46.6% of the eastern Indians tested had the haplogroup A.

## Discussion

The average number of CAG repeats in the normal individuals is reported to be 17 to 20 18. The commonest CAG repeat in the present sample is 18, and the mean CAG repeat size is 18.3, which is modestly higher than that found in populations from Africa (16.2), Japan (16.9) and China (17.0); but closer to that found in European (17.8) population 3 ^,^ 19 suggesting a genetic structure at this locus closer to the European population. Interestingly, the tribal population seems to have the lowest number of CAG repeats, with absence of any repeats greater than 18 10. This is perhaps consistent with lower repeat sizes observed in older populations (e.g sub-Saharan African populations).

The size of CAG repeats are evolutionarily controlled expansions which might benefit neuro-developmental processes 20, and may be conserved in the healthy population. The mechanism of transition from normal CAG to pathogenic expanded CAG can be envisaged by stepwise occurrence of intermediate CAG repeats followed by the genetic anticipation which would drive them into the pathological range of > 39 CAG repeats 21. This is reflected in the large 40-50 CAG repeat group which constitutes the majority (84%) of HD samples with late AAO (40.2 years). Thus the multistep increase in CAG number appears to play a role in CAG expansion in Indian population.

The CAG expansions beyond 50 CAG (N=16) constituted a smaller group, and had, correspondingly, a more severe illness phenotype with an early AAO (19.5 years). This is similar to those reported in other samples 22 ^,^ 23 ^,^ 24. The mechanisms of the CAG expansion, and abnormal folding of mutant Htt leading to neurodegeneration need to be explored further using animal and cellular models 7 ^,^ 25 ^,^ 26 ^,^ 27. Allele specific associations were not observed for the (CCG)_7 _or (CCG)_10_ 10 and ∆2642 deletion was found to be over-represented on expanded HTT alleles 14 ^,^ 28 ^,^ 29. The present study validates previous findings. Thus the underlying genetic diversity of HTT alleles needs to be addressed in south Asian populations.

In this sample, based on the three SNPs (rs762855- rs3856973- rs 4690073), haplogroup A was most common which corresponds to the Caucasian HD haplogroup 3. In addition, individuals from different parts of India shared this haplogroup, indicating that it is not a region specific effect. Sequencing the region further to permit finer mapping of these haplogroups is essential to comment upon the origin and spread of this mutation in India.

In summary, more than a 100 individuals have tested positive for HD at this centre, and this implies almost a 1000 subjects at risk (including siblings and children). The current population of India is estimated to be 1.27 billion. There is no population survey based data on the prevalence of HD from India. Extrapolating from the prevalence reported in south Asian population in UK 8 ; HD prevalence = 15.9 per million), or a more reasonable estimate of around 30/million) it can be speculated that between 20,000-40,000 people, are suspected to be affected with HD in Indian population, and more than 0.2 million may be at risk". It is suggested that the prevalence of HD in India should not be underestimated, since the occurrence of HD in India was proposed to be higher within the Asian population 3 . Therefore, establishing an Indian HD registry in a combined effort with diagnostic centers across the country and development of special care services in India needs to be considered. Based on HD associations from western countries, the average cost for genetic confirmation is about 1,000 USD/sample and the average annual cost of the medical service per HD patient is estimated to be 10,500-40,000 USD. Thus a considerable investment in clinical care, as well as family and social care services needs to be resolved in India. Though these are ‘rare’ syndromes, it is essential that we try to understand the genetic and clinical progression of this ‘single’ gene disorder with complex consequences and develop services focused to address the various problems the patients and their families face at different stages.

## Corresponding author

Dr Mahesh Kandasamy PhD, Research Scientist, Molecular Genetics Laboratory, Neurobiology Research Centre, Department of Psychiatry, National Institute of Mental Health and Neurosciences (NIMHANS), Hosur Road, Bangalore, 560 029, Karnataka, INDIA. Email: pkmahesh5@gmail.com, Tel. No. +91 80 26995263

## Keywords

Huntington's disease, HTT, CAG repeats, Ethnicity, Haplotype, India

## References

[1] Allitto BA, McClatchey AI, Barnes G, Altherr M, Wasmuth J, Frischauf AM, MacDonald ME, Gusella J. Assay by polymerase chain reaction (PCR) of multi-allele polymorphisms in the Huntington's disease region of chromosome 4. Mol Cell Probes. 1992 Dec;6(6):513-20. PubMed PMID:1480191. 1480191 10.1016/0890-8508(92)90048-3

[2] Walker FO. Huntington’s disease. Lancet. 2007;369(9557):218-228. doi:10.1016/S0140-6736(07)60111-1. 10.1016/S0140-6736(07)60111-1 17240289

[3] Warby SC, Visscher H, Collins JA, et al. HTT haplotypes contribute to differences in Huntington disease prevalence between Europe and East Asia. Eur J Hum Genet. 2011;19(5):561-566. doi:10.1038/ejhg.2010.229. 10.1038/ejhg.2010.229 PMC3083615 21248742

[4] ACMG/ASHG statement. Laboratory guidelines for Huntington disease genetic testing. The American College of Medical Genetics/American Society of Human Genetics Huntington Disease Genetic Testing Working Group. Am J Hum Genet. 1998;62(5):1243-1247. PMC1377103 9545416

[5] Wexler NS, Lorimer J, Porter J, et al. Venezuelan kindreds reveal that genetic and environmental factors modulate Huntington’s disease age of onset. Proc Natl Acad Sci USA. 2004;101(10):3498-3503. doi:10.1073/pnas.0308679101. 10.1073/pnas.0308679101 PMC373491 14993615

[6] Roos RAC. Huntington’s disease: a clinical review. Orphanet J Rare Dis. 2010;5(1):40. doi:10.1186/1750-1172-5-40 10.1186/1750-1172-5-40 PMC3022767 21171977

[7] Squitieri F, Andrew SE, Goldberg YP, et al. DNA haplotype analysis of Huntington disease reveals clues to the origins and mechanisms of CAG expansion and reasons for geographic variations of prevalence. Hum Mol Genet. 1994;3(12):2103-2114. 10.1093/hmg/3.12.2103 7881406

[8] Shiwach RS, Lindenbaum RH. Prevalence of Huntington’s disease among UK immigrants from the Indian subcontinent. Br J Psychiatry. 1990;157:598-599. 10.1192/bjp.157.4.598 2151860

[9] Khosla SN, Arora BS. Huntington’s chorea (a clinical study). J Assoc Physicians India. 1973;21(2):247-250. 4275868

[10] Pramanik S, Basu P, Gangopadhaya PK, et al. Analysis of CAG and CCG repeats in Huntingtin gene among HD patients and normal populations of India. Eur J Hum Genet. 2000;8(9):678-682. doi:10.1038/sj.ejhg.5200515. 10.1038/sj.ejhg.5200515 10980573

[11] Murgod UA, Saleem Q, Anand A, Brahmachari S., Jain S, Muthane UB. A clinical study of patients with genetically confirmed Huntington’s disease from India. Journal of the Neurological Sciences. 2001;190(1–2):73-78. doi:10.1016/S0022-510X(01)00593-7. 10.1016/s0022-510x(01)00593-7 11574110

[12] Saleem Q, Muthane U, Verma IC, Brahmachari SK, Jain S. Expanding colonies and expanding repeats. The Lancet. 2002;359(9309):895-896. doi:10.1016/S0140-6736(02)07941-2. 10.1016/S0140-6736(02)07941-2 11897323

[13] Harper PS. The epidemiology of Huntington’s disease. Hum Genet. 1992;89(4):365-376. 10.1007/BF00194305 1535611

[14] Saleem Q, Roy S, Murgood U, et al. Molecular analysis of Huntington’s disease and linked polymorphisms in the Indian population. Acta Neurol Scand. 2003;108(4):281-286. 10.1034/j.1600-0404.2003.00133.x 12956863

[15] Miller SA, Dykes DD, Polesky HF. A simple salting out procedure for extracting DNA from human nucleated cells. Nucleic Acids Res. 1988;16(3):1215. 10.1093/nar/16.3.1215 PMC334765 3344216

[16] Vasilief I. QtiPlot - Data Analysis and Scientific Visualisation Version 0.9.8.4.; 2011. Available at: http://soft.proindependent.com/qtiplot.html.

[17] Stephens M, Smith NJ, Donnelly P. A new statistical method for haplotype reconstruction from population data. Am J Hum Genet. 2001;68(4):978-989. doi:10.1086/319501. 10.1086/319501 PMC1275651 11254454

[18] Myers RH. Huntington’s disease genetics. NeuroRx. 2004;1(2):255-262. doi:10.1602/neurorx.1.2.255. 10.1602/neurorx.1.2.255 PMC534940 15717026

[19] Warby SC, Visscher H, Butland S, Pearson CE, Hayden MR. Response to Falush: A Role for cis-Element Polymorphisms in HD. Am J Hum Genet. 2009;85(6):942-945. doi:10.1016/j.ajhg.2009.11.006. 10.1016/j.ajhg.2009.11.006 PMC2790565 20004773

[20] Nithianantharajah J, Hannan AJ. Dynamic mutations as digital genetic modulators of brain development, function and dysfunction. Bioessays. 2007;29(6):525-535. doi:10.1002/bies.20589. 10.1002/bies.20589 17508392

[21] Costa M do C, Magalhães P, Guimarães L, Maciel P, Sequeiros J, Sousa A. The CAG repeat at the Huntington disease gene in the Portuguese population: insights into its dynamics and to the origin of the mutation. J Hum Genet. 2006;51(3):189-195. doi:10.1007/s10038-005-0343-8. 10.1007/s10038-005-0343-8 16372132

[22] Gatto E, Parisi V, Persi G, et al. Clinical and genetic characteristics in patients with Huntington’s Disease from Argentina. Parkinsonism Relat Disord. 2012;18(2):166-169. doi:10.1016/j.parkreldis.2011.09.011. 10.1016/j.parkreldis.2011.09.011 21962718

[23] Brinkman RR, Mezei MM, Theilmann J, Almqvist E, Hayden MR. The likelihood of being affected with Huntington disease by a particular age, for a specific CAG size. Am J Hum Genet. 1997;60(5):1202-1210. PMC1712445 9150168

[24] Lee J-M, Ramos EM, Lee J-H, et al. CAG repeat expansion in Huntington disease determines age at onset in a fully dominant fashion. Neurology. 2012;78(10):690-695. doi:10.1212/WNL.0b013e318249f683. 10.1212/WNL.0b013e318249f683 PMC3306163 22323755

[25] McMurray CT. Mechanisms of trinucleotide repeat instability during human development. Nat Rev Genet. 2010;11(11):786-799. doi:10.1038/nrg2828. 10.1038/nrg2828 PMC3175376 20953213

[26] Møllersen L, Rowe AD, Larsen E, Rognes T, Klungland A. Continuous and Periodic Expansion of CAG Repeats in Huntington’s Disease R6/1 Mice. PLoS Genet. 2010;6(12):e1001242. doi:10.1371/journal.pgen.1001242. 10.1371/journal.pgen.1001242 PMC3000365 21170307

[27] Petruska J, Hartenstine MJ, Goodman MF. Analysis of strand slippage in DNA polymerase expansions of CAG/CTG triplet repeats associated with neurodegenerative disease. J Biol Chem. 1998;273(9):5204-5210. 10.1074/jbc.273.9.5204 9478975

[28] Lucotte G, Gérard N, Roubertoux P, Schmitt I, Riess O. Relationships of the 2642 deletion polymorphism (delta 2642) in the huntingtin gene with the CAG repeat expansion length and age at onset of the disease. Genet Couns. 1996;7(4):297-302. 8985734

[29] Almqvist E, Spence N, Nichol K, et al. Ancestral differences in the distribution of the delta 2642 glutamic acid polymorphism is associated with varying CAG repeat lengths on normal chromosomes: insights into the genetic evolution of Huntington disease. Hum Mol Genet. 1995;4(2):207-214. 10.1093/hmg/4.2.207 7757069

